# Sin Nombre Virus Infection in Deer Mice, Channel Islands, California

**DOI:** 10.3201/eid1412.080935

**Published:** 2008-12

**Authors:** John L. Orrock, Brian F. Allan

**Affiliations:** Washington University, St. Louis, Missouri, USA

**Keywords:** hantavirus, rodent, Sin Nombre virus, letter

**To the Editor:** Sin Nombre virus (SNV) is a highly virulent strain of hantavirus associated with rodent hosts in North America (*1*,*2*). Documenting the prevalence of SNV in wild rodent populations is an important component of determining risk for exposure and ultimately providing sound recommendations for epidemiologic management (*3*). Prevalence of SNV is highly variable. Deer mice (*Peromyscus maniculatus*) that inhabit the Channel Islands off the California coast often have rates of SNV that greatly exceed values on the mainland (*2*). Even though these islands have high rates of SNV prevalence and are recreational areas for humans, no surveys of the Channel Islands have been performed to document the dynamics of prevalence since 1994–1996 (*2*,*4*). We visited 4 of the Channel Islands in 2007 to document rates of SNV prevalence in *P. maniculatus*.

From May 3–15, 2007, we visited 4 of the Channel Islands off the California coast: East Anacapa Island, Santa Barbara Island, San Miguel Island, and Santa Rosa Island. On each island, mice were captured by using Sherman live traps from habitats characterized by giant coreopsis (*Coreopsis gigantea*), a shrub native to California, to provide a standardized habitat for comparisons across islands. The number of sampling areas depended largely upon the distribution of *C*. *gigantea* habitat and logistical considerations during each island visit (Table). Upon capture of the mice, blood samples were taken from the submandibular vein by using Medi-Point animal lancets (Medi-Point International, Inc., Mineola, NY, USA) and stored in sterile micropipette tubes. Samples were stored on ice until shipment to the California Department of Health Services’ Viral and Rickettsial Disease Laboratory for processing. *P*. *maniculatus* serum samples were examined for immunoglobulin (Ig) G antibodies to the SNV nucleocapsid protein by ELISA with Centers for Disease Control and Prevention reagents (*5*).

**Table:** Sin Nombre virus in *Peromyscus maniculatus* mice on 4 Channel Islands, California, May 3–15, 2007*

Island*	No. trap nights	Prevalence, %	
		2007	1994
East Anacapa†	180	0	0
San Miguel‡	104	26.3	17.9
Santa Barbara§	216	0	0
Santa Rosa¶	216	47.6	58

*The number of captured mice that were sampled for Sin Nombre virus (SNV) was 23 on East Anacapa, 19 on San Miguel Island, 15 on Santa Barbara Island, and 21 on Santa Rosa Island. The 1994 data in the table are from a study by Jay et al. (*2*) and are included for comparison purposes. †East Anacapa: 34º00'56"N/119º21'49"W. ‡San Miguel: 34º02'18"N/120º20'54"W. §Santa Barbara: 33º28'30"N/119º02'12"W. ¶Santa Rosa: 34º00'03"N/120º03'30”W.

Detailed information regarding SNV prevalence, sampling location, and sampling effort is presented in the Table. We compare our 2007 data with data collected in 1994 by Jay et al. (*2*) because 1994 was the only other year when all 4 islands used in our study were sampled. Graham and Chomel (*4*) also collected data from San Miguel Island and Santa Rosa Island in 1995 and 1996 (the use of the average prevalence from 1995 and 1996 for these 2 islands does not change any of our results).

There was no significant difference in prevalence of SNV antibodies between our 2007 results and the prevalence found by Jay et al. (*2*) in 1993–1994 (paired *t* test *t* = 0.13, 3; df = 3; p = 0.91). Overall, 36 male and 42 female mice were captured; the sex of captured animals was independent of SNV infection (9 males and 6 females positive for SNV; test of independence χ^2^ = 0.28, 1 df, p = 0.59). We captured only 2 subadult mice on islands where we also detected antibodies to SNV; 1 mouse tested positive, the other tested negative. Although our sample sizes precluded detecting very low rates of SNV infection with confidence on Santa Barbara and East Anacapa Islands, the consistency of our results with those of Jay et al. (*2*) suggests that our sampling was sufficient for comparative purposes.

Several studies now indicate the importance of long-term surveillance of SNV prevalence in wild rodent populations for understanding the factors that may contribute to outbreaks of human disease, e.g (*6*). These studies often document the generally positive, though often temporally delayed, relationship between population density of *P. maniculatus* and seroprevalence for SNV (*7*). Our results suggest a high degree of temporal stability in prevalence of antibodies to SNV in *P. maniculatus* on the Channel Islands, despite considerable variation in host population density between earlier studies and ours (*4*,*8*). Although we cannot know the prevalence of SNV among *P. maniculatus* on the Channel Islands in periods between the studies by Jay et al. (*2*), Graham and Chomel (*4*), and our own, SNV prevalence on these islands is quite similar to levels previously recorded both for islands with relatively low prevalence (i.e., East Anacapa and Santa Barbara Islands) or high prevalence (i.e., San Miguel and Santa Rosa Islands).

Future studies comparing long-term dynamics on islands and related mainlands are needed to examine the possibility that insular systems provide unique opportunities to understand the factors affecting pathogen dynamics and human risk. Given the substantial variation in mouse population density among different habitats within these islands and variation in prevalence among trapping areas in our study (Table) and others (*4*), we also recommend that future studies focus on the diverse array of habitats where *P. maniculatus* is found on the islands to more completely characterize within-island risk.
